# Flavonoid Extract from Propolis Provides Cardioprotection following Myocardial Infarction by Activating PPAR-*γ*

**DOI:** 10.1155/2022/1333545

**Published:** 2022-07-05

**Authors:** Qian Wang, Rui Chen, Qian Tang, Jin-ying Chen, Yan Wang, Dian-jun Sui, Ai-dong Liu

**Affiliations:** ^1^The Third Affiliated Hospital of Changchun University of Traditional Chinese Medicine, Changchun, China; ^2^Changchun University of Traditional Chinese Medicine, Changchun, China

## Abstract

We have previously reported that flavonoid extract from propolis (FP) can improve cardiac function in rats following myocardial infarction (MI). However, the mechanisms responsible for the cardioprotective effects of FP have not been fully elucidated. In the current study, we explored whether FP can reduce inflammatory cytokines and attenuate sympathetic nerve system activity and antiendoplasmic reticulum (ER) stress and whether the cardioprotective effects are related to peroxisome proliferator-activated receptor gamma (PPAR-*γ*) activation. Sprague Dawley rats were randomly divided into six groups: Sham group received the surgical procedure but no artery was ligated; MI group received ligation of the left anterior descending (LAD) branch of the coronary artery; MI + FP group received FP (12.5 mg/kg/d, intragastrically) seven days prior to LAD ligation; FP group (Sham group + 12.5 mg/kg/d, intragastrically); MI + FP + GW9662 group received FP prior to LAD ligation with the addition of a specific PPAR-*γ* inhibitor (GW9662), 1 mg/kg/d, orally); and MI + GW9662 group received the PPAR-*γ* inhibitor and LAD ligation. The results demonstrated that the following inflammatory markers were significantly elevated following MI as compared with expression in sham animals: IL-1*β*, TNF-*α*, CRP; markers of sympathetic activation: plasma norepinephrine, epinephrine and GAP43, nerve growth factor, thyroid hormone; and ER stress response markers GRP78 and CHOP. Notably, the above changes were attenuated by FP, and GW9662 was able to alleviate the effect of FP. In conclusion, FP induces a cardioprotective effect following myocardial infarction by activating PPAR-*γ*, leading to less inflammation, cardiac sympathetic activity, and ER stress.

## 1. Introduction

Many cardiovascular diseases can lead to deterioration of ventricular shape, size, and function, contributing to negative ventricular remodeling [1]. Ventricular remodeling after myocardial infarction is a leading cause of cardiac dysfunction [2]. The expression of inflammatory factors, including tumor necrosis factor-*α* (TNF-*α*) and interleukin (IL)-1*β*, significantly increases after myocardial infarction (MI) in rats and has important roles in the etiology of ventricular remodeling [3]. With respect to cardiac remodeling, these inflammatory cytokines promote the expression and activation of matrix metalloproteinase which lead to post-MI extracellular matrix deposition and degradation (antioxidant nanomaterials in advanced diagnoses and treatments of ischemia reperfusion injuries) [4, 5]. Inhibition of the inflammatory response markedly reduces left ventricular endstolic dimension (LVESD), left ventricular end-diastolic dimension (LVEDD), and left ventricular end-diastolic pressure while increasing fractional shortening and dP/dt [6].

Previous studies have demonstrated that abnormal hemodynamics post-MI can induce excess sympathetic adrenergic system activation. This aberrant sympathetic nerve terminal activity leads to spillover release of adrenaline and norepinephrine and concomitant alteration in ventricular architecture [7]. Soeki T. et al. reported that ghrelin can improve myocardial fibrosis and cardiac function in rats with myocardial infarction via attenuation of cardiac sympathetic activity [8]. Further, others have shown that *β*-adrenergic blockade could counteract adverse ventricular remodeling by inhibition of the sympathetic nervous system [9]. Lastly, ER stress has been implicated in the pathophysiology of cellular fibrosis and apoptosis [10, 11], such that attenuation of ER stress prevents post-infarction-induced cardiac rupture and remodeling [12].

Propolis is a dietary supplement derived from a resinous hive product collected by honeybees from various exudates of trees and plants [13]. Propolis has been shown to possess protective effects against hypertension [14] and atherosclerosis [15]. Flavonoids are major functional components of propolis, which have a long history of therapeutic use. Due to their activity on neurotransmitters and hormones, antioxidant effects, and enzymatic modulation, flavonoids have been used to treat a variety of diseases [16].

Our research group has successfully extracted flavonoids from propolis (FP) and identified the major active components in FP, including 12-acetoxyviscidone, isosakuranetin, galangin, kaempferol, pinobanksin-3-O-acetate, chrysin, and pinocembrin. Our prior work has demonstrated that FP is cardioprotective against isoproterenol-induced pathological cardiac hypertrophy in mice [17] and attenuates cardiac fibrosis and oxidative stress markers in a rat MI model [18]. However, it is still unclear how FP may affect inflammatory cytokines, cardiac sympathetic activity, and endoplasmic reticulum stress in a rat model of MI.

## 2. Materials and Methods

### 2.1. FP Extraction

The FP specimen was extracted and analyzed as previously reported [17]. Briefly, FP was ground into powder and dissolved in 10x of 75% ethanol/water to extract the active flavonoids. The solution was filtered using a 0.45 *μ*m membrane (Pall Corporation, ^#^66585; Shanghai, China). The filter was dried and the extract was subsequently dissolved in a solution of 10 : 1 chloroform : ethanol and 1% NaOH solution (using the same volume as chloroform: ethanol). The solvent was adjusted to pH 6 using 1% HCl and then filtered and dried to collect the FP product.

### 2.2. Rat MI Model

All experiments were conducted in accordance with the Guide for the Care and Use of Laboratory Animals published by the U.S. National Institute of Health (NIH Publication No. 85-23, revised 1996). Male Sprague Dawley (SD) rats (8 weeks old, 210–260 g) were housed in the Experimental Animal Center of Jilin University. The rats were randomly divided into six groups: Sham group, MI group, MI + FP group (12.5 mg/kg/d, intragastrically, seven days prior to LAD ligation through termination of the animal at day 28), FP group, MI + FP + GW9662 (1 mg/kg/d, orally, simultaneously with FP intragastrically) group, and MI + GW9662 group. To induce MI, following anesthetization with pentobarbital sodium (50 mg/kg), an incision was made at the fourth intercostal space and the left coronary artery was ligated with 6-0 suture. In Sham animals, the suture was passed under the artery but was not secured to induce ligation.

### 2.3. Echocardiographic Measurements

Left ventricular (LV) function was evaluated using transthoracic echocardiography (Visualsonics Toronto, Ontario, Canada). 28 days after the index procedure M-mode tracing was used to calculate LV ejection fraction (LVEF), fractional shortening (FS), LVEDD, and LVESD.

### 2.4. Myocardial Histopathology

The rats were sacrificed 28 days after the index procedure and hearts were isolated for histological analysis. Masson's trichrome staining was performed as previously reported [18]. Collagen was dyed blue, and cardiomyocytes were dyed red. Myocardial fibrosis was analyzed using ImagePro Plus 6.0 software (Media Cybernetics, Rockville, MD, USA). The width of the thinnest part of the infarct defined the infarction wall thickness. Infarct size was calculated as the infarct wall area divided by LV area.

### 2.5. Epinephrine, Norepinephrine, and Dopamine Quantification

Plasma catecholamines, including epinephrine, norepinephrine, and dopamine, were quantified by high performance liquid chromatography connected to an electrochemical detector according to the standard techniques using a modified procedure as previously reported [19]. An Atlantis dC18 column (Massachusetts, USA) was used to separate catecholamines, and 3,4-dihydroxybenzylamine was used as the internal standard. Briefly, an antioxidant sodium metabisulfite (0.125 mM) and 3,4-dihydroxybenzylamine were added to 0.25 mL of plasma, followed by incubation for 0.5 h. The samples were then centrifuged (5000 rpm for 5 minutes at room temperature), the supernatant was discarded, and the column was washed. The catecholamines were eluted with 80 mL of 0.1 N HCl supplemented with 0.1 mmol/L sodium metabisulfite. Empower software (Multiskan MK3, Thermo) was used to analyze the data and catecholamine levels were normalized by recovery of the internal standard.

### 2.6. Hydroxyproline and Plasma NT-pro-BNP, BNP, Angiotensin II, and CRP Assays

Commercial kits (Nanjing Jiancheng Bioengineering Institute, Nanjing, China; Shang Hai Ze Ye Biotechnology Co., Ltd, Shang Hai, China) were used to assay hydroxyproline content of the LV myocardium as well as plasma NT-pro-BNP, BNP, CRP, and angiotensin II content according to the manufacturer's instructions.

### 2.7. Quantitative RT-PCR

Total miRNAs in the left ventricle were extracted using Trizol reagent (Invitrogen, USA), according to the manufacturer's instructions. Reversion into cDNA was performed using Taqman MicroRNA Reverse Transcription Kit (TaKaRa, Dalian, China). The protocol is as follows: 1 cycle of 95°C for 10 min, 40 cycles of 95°C for 30 s, and 60°C for 1 min; 1 cycle of 95°C for 1 min, 55°C for 30 s, and 95°C for 30 s. The GAPDH gene was used as the reference gene. Primer sequences used for quantitative RT-PCR are listed in Table 1.

### 2.8. Western Blotting

Western blotting was used to analyze protein expression. Samples were obtained from left ventricle myocardial tissue, and protein concentration was assayed by a bicinchoninic acid protein assay kit (Pierce, USA). Total protein (50 *μ*g per sample) was loaded on 10% SDS-PAGE gels and transferred onto polyvinylidene fluoride (PVDF) membranes. Following blocking with 5% nonfat milk, the PVDF membranes were incubated with primary antibodies against nerve growth factor (NGF; Abcam, Cat. No. ab52918, 1 : 1000), thyroid hormone (TH; Abcam, Cat. No. ab137869, 1 : 5000), growth associated protein 43 (GAP43; Abcam, Cat. No. ab16053, 1 : 1000), 78-kDa glucose-regulated protein (GRP 78; Santa Cruz, Cat. No. sc-376768, 1 : 1000), C/EBP homologous protein (CHOP; Cell Signaling Technology, Cat. No. 2895, 1 : 1000) at 4°C overnight, followed by secondary antibody incubated for 1.5 h at room temperature. Images were developed using an enhanced chemiluminescence substrate (ECL Plus, GE Healthcare, United States) for 1 min in the dark. Glyceraldehyde-3-Phosphate Dehydrogenase (GAPDH; ABclonal, Cat. No. AC001, 1 : 1000) was used as an internal loading control.

### 2.9. Statistical Analysis

Normally distributed experimental data were expressed as mean ± standard error of the mean (SEM). Statistical significance was analyzed by one-way ANOVA followed by Bonferroni's post hoc test. SPSS 17.0 statistical software (SPSS Inc., Chicago, IL, USA) and GraphPad Prism 6 (GraphPad Sofware Inc., La Jolla, CA, USA) were used for statistical analysis and graphing. *P* < 0.05 was considered statistically significant.

## 3. Results

### 3.1. Effect of FP on Myocardial Histopathology and Cardiac Function following MI in Rats

There was no significant difference in infarct size between the different groups. However, the ventricular wall thickness and the body weights were significantly decreased in the MI group compared with the Sham group. Interesting, the ventricular wall thickness and the body weights were significantly higher in MI + FP group than that of the MI group. There was no statistical difference in the ventricular wall thickness and the body weights of animals between the Sham and FP groups. The lung wet/dry wet ratio was significantly higher in the MI group compared with Sham animals, while the ratio was significantly lower in MI + FP animals compared to the MI group. A similar effect was also observed in liver wet/dry wet ratio. There were no significant differences in lung wet/dry wet ratio and liver wet/dry wet ratio between Sham or FP groups. Notably, the effect of FP on organ weights and dimensions was attenuated by GW9662.

Measures of LV function were used to define cardiac performance. The results demonstrated that LVEF and LVFS significantly decreased in MI rats compared with Sham groups. Measures of LVEF and LVFS were significantly higher in the MI + FP group compared with the MI group. There were no significant differences in LVEF or LVFS between the Sham and FP groups. LVID (d) and LVID (s) significantly increased in MI rats compared with Sham controls, while LVID (d) and LVID (s) significantly decreased in the MI + FP group compared with the MI group. There were no significant differences in LVID (d) or LVID (s) between the Sham and FP groups. Notably, the protective effects of FP on measures of cardiac function were alleviated by GW9662 (Table 2).

### 3.2. Effect of FP on PPAR-*γ* mRNA and Protein Expression in a Rat MI Model

**Figure 1:**
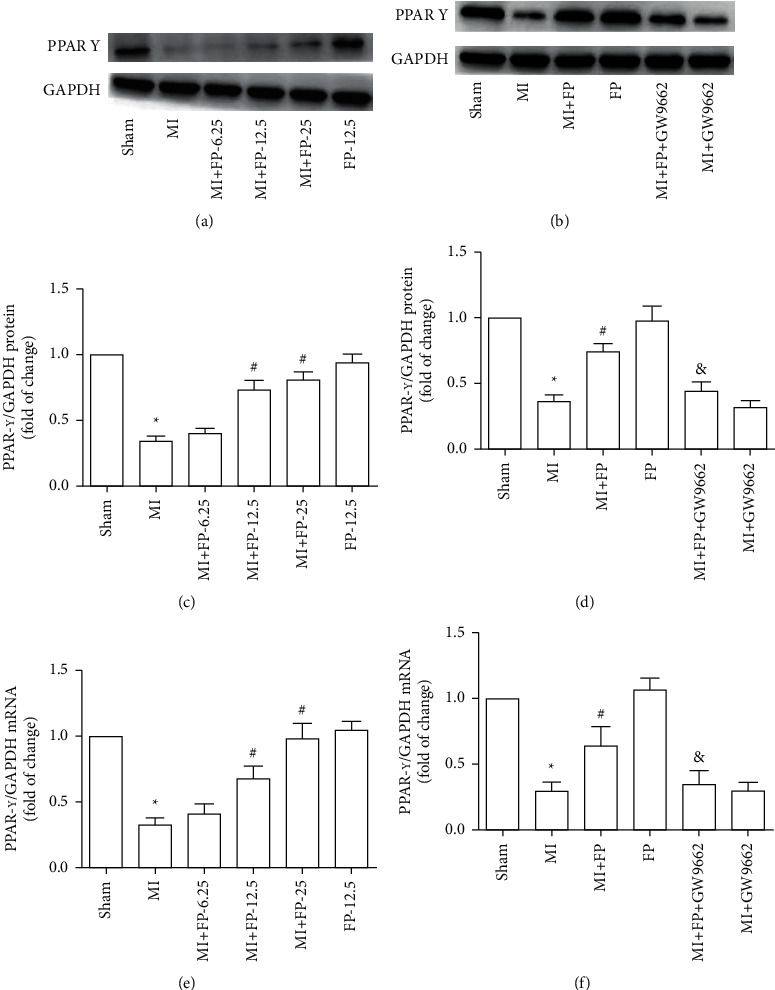
The effect of FP on PPAR-*γ* protein and mRNA expression in MI rats. (a) PPAR-*γ* protein expression at different concentrations of FP (6.25, 12.5, and 25 mg/kg/d) in rats from the MI group. (b) PPAR-*γ* protein expression in the Sham, MI, MI + FP, FP, MI + FP + GW9662, and MI + GW9662 groups. (c) Quantitative analysis of (a). (d) Quantitative analysis of (b). (e) PPAR-*γ* mRNA expression at different concentrations of FP (6.25, 12.5, and 25 mg/kg/d) in rats from the MI group. (f) PPAR-*γ* mRNA expression in the Sham, MI, MI + FP, FP, MI + FP + GW9662, and MI + GW9662 groups. The results are expressed as the mean ± SEM; *n* = 9 per group; ^*∗*^*P* < 0.05 versus the Sham group; ^#^*P* < 0.05 versus the MI group; ^&^*P* < 0.05 versus the MI + FP group.

The current experimental results show that PPAR-*γ* mRNA and protein expression were markedly decreased in the MI group compared with the Sham group. FP (12.5 or 25 mg/kg/d, but not 6.25 mg/kg/d) upregulated PPAR-*γ* mRNA and protein expression; however, the effect of FP was reversed by GW9662. There were no significant differences in PPAR-*γ* mRNA and protein expression between the Sham and FP groups (Figure 1).

### 3.3. Effect of FP on Cardiac Fibrosis in a Rat MI Model

**Figure 2:**
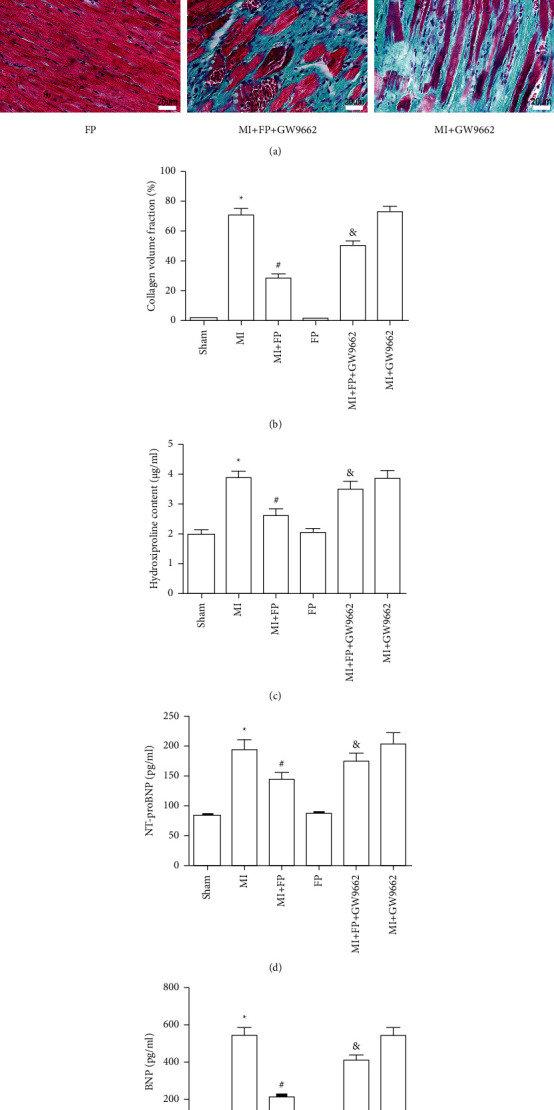
The effect of FP on cardiac fibrosis and hydroxyproline, NT-pro-BNP, and BNP content in the Sham, MI, MI + FP, FP, MI + FP + GW9662, and MI + GW9662 groups. (a) Masson stain showing changes in fibrosis across experimental groups. (b) Quantitative analysis of (a). (c) ELISA method assessing hydroxyproline content. (d) ELISA method assessing NT-pro-BNP content. (e) ELISA method assessing BNP content. The results are expressed as the mean ± SEM; *n* = 9 per group; ^*∗*^*P* < 0.05 versus the Sham group; ^#^*P* < 0.05 versus the MI group; and^&^*P* < 0.05 versus the MI + FP group.

Since myocardial fibrosis is a main pathological contributor to ventricular remodeling, the cardiac collagen fraction was assessed across experimental groups [20]. The results demonstrated that cardiac collagen volume was significantly elevated in MI rats compared to the Sham group. However, cardiac collagen volume was markedly lower in the MI + FP group compared with the MI group. Notably, the effect of FP on collagen volume was reversed by GW9662. There were no significant differences in cardiac collagen fraction between the Sham and FP groups, or between the MI and MI + GW9662 groups. The same trends in collagen volume across groups were also observed in hydroxyproline content (Figures 2(a)–2(c)).

### 3.4. Effect of FP on Plasma Pro-BNP and BNP Content in a Rat Model of MI

To explore the effect of FP on markers of heart failure, we assayed plasma pro-BNP and BNP content in Sham, MI, MI + FP, FP, MI + FP + GW9662, and MI + GW9662 groups. The result showed that pro-BNP and BNP content were significantly increased in MI rats compared to Sham groups. However, pro-BNP and BNP content was markedly lower in the MI + FP group compared with the MI group. Notably, the effect of FP was reversed by GW9662. There were no significant differences in pro-BNP and BNP content between the Sham and FP groups, or between the MI and MI + GW9662 groups (Figures 2(d)–2(e)).

### 3.5. Effect of FP on IL-1*β* and TNF-*α* Expression and CRP Content in a Rat Model of MI

**Figure 3:**
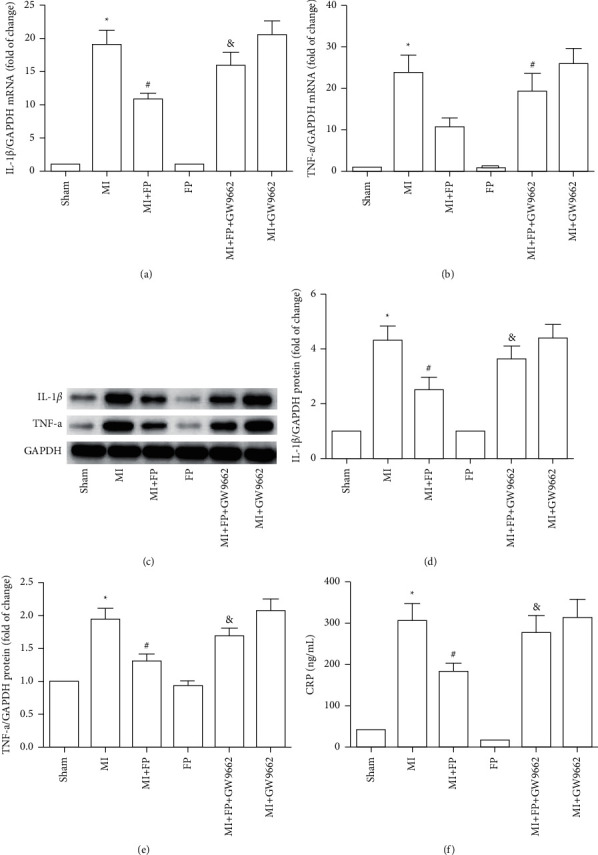
The effect of FP on IL-1*β*, TNF-*α* expression, and CRP content in the Sham, MI, MI + FP, FP, MI + FP + GW9662, and MI + GW9662 groups. (a) IL-1*β* mRNA were assayed by qRT-PCR. (b) TNF-*α* mRNA was assayed by qRT-PCR. (c) IL-1*β* and TNF-*α* protein expression levels were assayed by Western blot. (d-e) Quantitative analysis of (c). (f) ELISA method assessing CRP. The results are expressed as the mean ± SEM; *n* = 9 per group; ^*∗*^*P* < 0.05 versus the Sham group; ^#^*P* < 0.05 versus the MI group; ^&^*P* < 0.05 versus the MI + FP group.

To explore whether FP exhibits anti-inflammatory effects, we detected protein and mRNA expression of IL-1*β* and TNF-*α* cytokines in LV myocardium across experimental groups. The results demonstrated that TNF-*α* and IL-1*β* protein and mRNA expression significantly increased in the MI group compared with Sham animals. However, TNF-*α* and IL-1*β* protein and mRNA expression were markedly lower in the MI + FP group compared to the MI group. Notably, the effect of FP was reversed by GW9662. There were no significant differences in the protein and mRNA expression of these markers between Sham and FP group, or between the MI and MI + GW9662 groups. The same trends observed with these cytokines across groups were also observed in plasma CRP (Figure 3).

### 3.6. Effect of FP on Cardiac GAP43, NGF, and TH Protein Expression in a Rat Model of MI

**Figure 4:**
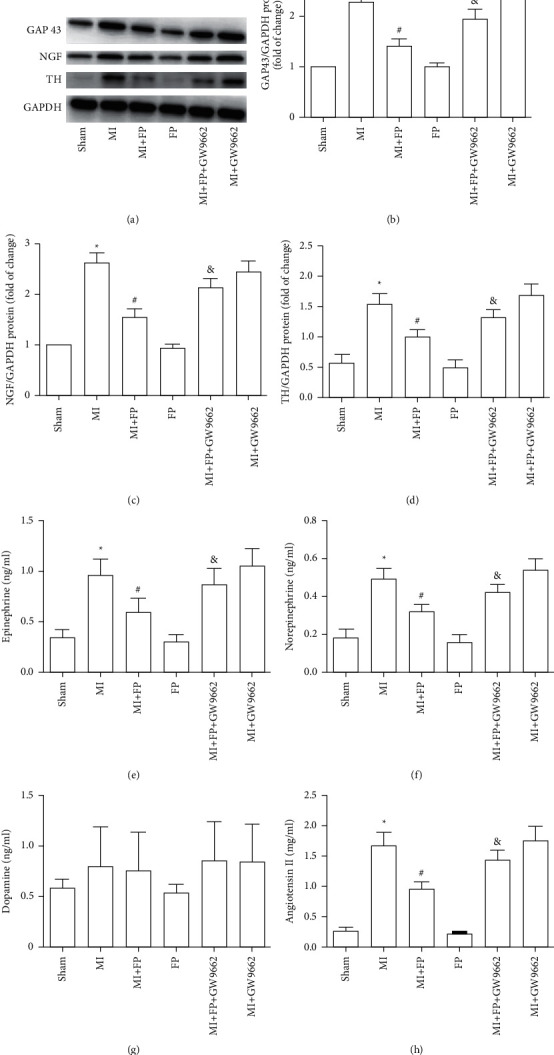
The effect of FP on GAP 43, NGF, and TH protein expression, and plasma epinephrine, norepinephrine, and dopamine content in the Sham, MI, MI + FP, FP, MI + FP + GW9662, and MI + GW9662 groups. (a) Representative Western blot images of GAP 43, NGF, and TH. (b–d) Quantitative analysis of (a). (e–h) ELISA method assessing epinephrine, norepinephrine, dopamine, and angiotensin II. The results are expressed as the mean ± SEM; *n* = 9 per group; ^*∗*^*P* < 0.05 versus the Sham group; ^#^*P* < 0.05 versus the MI group; ^&^*P* < 0.05 versus the MI + FP group.

To clarify whether the protective effects of FP on the myocardium of rats in the MI group were related to cardiac sympathetic activation, Western blot was used to detect GAP43, NGF, and TH protein expression. Our experiments showed that GAP43, NGF, and TH expression were significantly increased in the myocardium of rats in the MI group compared with the Sham group. GAP43, NGF, and TH protein expression levels were significantly lower in myocardium from the MI + FP group compared to tissue from the MI group. Notably, the protective effect of FP was reversed by GW9662. There were no significant differences in the expression of these proteins between Sham and FP groups, or between MI and MI + GW9662 groups (Figures 4(a)–4(d)).

### 3.7. Effect of FP on Plasma Catecholamine and Angiotensin II Concentrations in a Rat Model of MI

It is well accepted that catecholamines, including epinephrine, norepinephrine, and dopamine, are associated with ventricular remodeling after myocardial infarction [8]. We measured the effects of FP on epinephrine, norepinephrine, and dopamine concentrations across the experimental groups. The results showed that dopamine concentrations were not markedly different across groups. However, there was significant increase in epinephrine, norepinephrine, and angiotensin II concentrations in the MI group compared with Sham animals. Epinephrine, norepinephrine, and angiotensin II concentrations were markedly lower in MI + FP animals compared with the MI group. Notably, the effect of FP on catecholamines and angiotensin II was reversed by GW9662. There were no significant differences in plasma epinephrine, norepinephrine, or angiotensin II concentration between Sham and FP groups, or MI and MI + GW9662 groups (Figures 4(e)–4(h)).

### 3.8. Effect of FP on Cardiac GRP 78 and CHOP Expression in a Rat Model of MI

**Figure 5:**
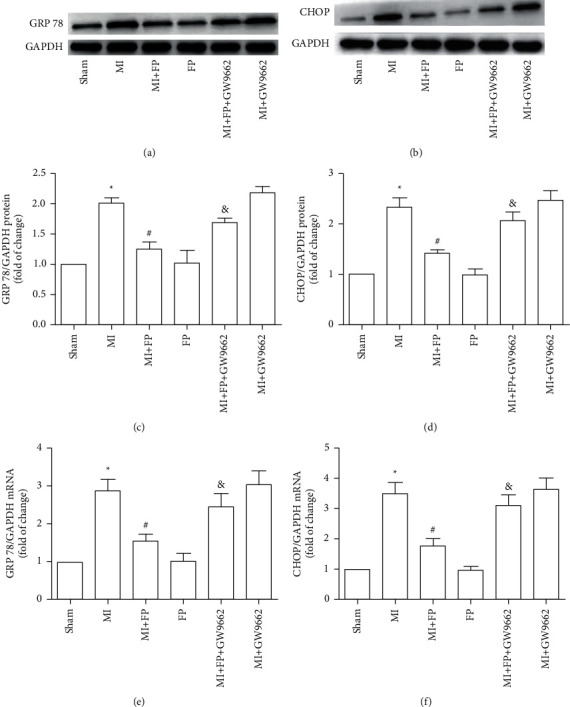
The effect of FP on GRP78 and CHOP expression in the Sham, MI, MI + FP, FP, MI + FP + GW9662, and MI + GW9662 groups. (a-b) Representative Western blot images of GRP78 and CHOP. (c-d) Quantitative analysis of (a-b). (e-f) GRP78 and CHOP mRNA were assayed by qRT-PCR. The results are expressed as the mean ± SEM; *n* = 9 per group; ^*∗*^*P* < 0.05 versus the Sham group; ^#^*P* < 0.05 versus the MI group; ^&^*P* < 0.05 versus the MI + FP group.

To determine whether the cardioprotective effects of FP involve modulation of endoplasmic reticulum stress, Western blot and RT-PCR were used to measure GRP 78 and CHOP protein and mRNA expression. The results demonstrated that GRP 78 and CHOP protein and mRNA expression were significantly increased in the myocardium of rats in the MI group compared with the Sham group. In contrast, GRP 78 and CHOP protein and mRNA expression were significantly lower in the myocardium of animals in the MI + FP group compared with the MI group. Notably, the effect of FP on markers of endoplasmic reticulum stress was reversed by GW9662. There were no significant differences in the expression of these proteins between the Sham and FP groups, and there were no differences between the MI and MI + GW9662 groups (Figure 5).

## 4. Discussion

The principle findings of the present study in a rat model of MI are as follows: (1) FP treatment decreased cardiac dysfunction and measures of myocardial remodeling, such as infarction wall thickness, heart weight, lung wet, liver wet, LVID(d), and LVID(s); (2) FP treatment decreased measures of inflammation, including plasma CRP and IL-1*β* and TNF-*α* mRNA and protein expression; (3) FP treatment decreased plasma indicators of sympathetic nerve activation, including GAP43, NGF, TH, epinephrine, and norepinephrine; (4) FP treatment decreased markers of endoplasmic reticulum stress, including GRP 78 and CHOP protein and mRNA expression; (5) the cardioprotective effects of FP mentioned above were attenuated by PPAR-*γ* inhibitor GW9662. Taken together, the protective effects of FP are mediated through PPAR-*γ* to decrease inflammatory cytokines, cardiac sympathetic activity, and endoplasmic reticulum stress.

Propolis, also known as bee glue, has previously been reported to exhibit pharmacological actions, including cardioprotective effects [15]. Propolis contains a variety of active ingredients, among which flavonoids are the most biologically active components [14]. Prior work has shown that FP can attenuate pathological cardiac hypertrophy and collagen deposition in isoproterenol-treated mice through inhibition of the PI3K/AKT signaling pathway [17]. Our research group has shown that FP can inhibit proliferation and migration of cardiac fibroblasts, stimulated by angiotensin II, through upregulation of SIRT1 [18]. However, previous experiments did not assess the effect of FP on inflammatory and sympathetic activity, which play important roles in myocardial remodeling in a rat model of MI.

Pathological changes after MI include alteration in myocardial structure, induction of oxidative stress, and ischemic stimulus contributing to the release of inflammatory factors [21]. This inflammatory response can result in both myocardial remodeling and heart failure [22]. Li B et al. have reported that interleukin (IL)-1*β* and tumor necrosis factor (TNF)-*α* mRNA expression significantly increase in noninfarcted myocardium in a rat model of MI [23]. Reducing the expression of inflammatory factors has the potential to delay ventricular remodeling and improve cardiac function following MI and could be a promising therapeutic target for novel interventions [6].

Increasing evidence demonstrates that the renin-angiotensin-aldosterone system (RAAS) is activated in patients with MI [24]. Angiotensin II (Ang II) is an important active component RAS, playing an important role on myocardial oxidative stress, collagen formation, and impaired ejection fraction [25, 26]. Ang II has also been shown to upregulate inflammation in a mouse model of MI [27]. The current study shows that FP can decrease inflammatory cytokines IL-1*β* and TNF-*α*, plasma CRP, and Ang II following MI in rats.

Autonomic nerves include both sympathetic and parasympathetic innervation. Under normal physiologic conditions both regulate the cardiovascular system to maintain a dynamic equilibrium. Following MI, there is an imbalance of sympathetic and parasympathetic, characterized by increased sympathetic activity and reduced vagal activity, which may worsen the inflammatory response after an ischemic insult [28]. Further, sympathetic system activation has been shown to contribute to ventricular remodeling after MI [29, 30]. Li et al. demonstrated that maintaining the balance of cardiac sympathetic innervation is cardioprotective by restoring ejection fraction in a rat model of cardiac dysfunction [31]. Accumulating evidence shows that cardiac sympathetic nerve activity leads to heterogeneous nerve sprouting and sympathetic hyperinnervation, which may cause cardiac arrhythmia and even sudden death [32–34]. Plasma catecholamines, including epinephrine, norepinephrine, and dopamine, are synthesized by the adrenal glands, released from sympathetic nerve endings, and reflect sympathetic activation [35, 36]. Karlsberg RP reported that plasma norepinephrine concentration is one of the key factors influencing morbidity and mortality in patients affected by MI [37]. Nerve growth factor (NGF) has an important role in modulating sympathetic nerve structure and function, density of sympathetic innervation, and the formation and persistence of heart failure [38, 39]. GAP43, TH, epinephrine, norepinephrine, and dopamine levels also represent sympathetic activity and were used to complement NGF in the current study to explore the effect of FP on cardiac sympathetic tone [40–42]. The results show that FP decreased epinephrine, norepinephrine, NGF, GAP43, and TH levels, suggesting that FP inhibits excess sympathetic nerve activation following MI.

ER stress exists in the pathophysiology of many cardiovascular diseases including cardiac hypertrophy, myocardial ischemia–reperfusion injury, and diabetic cardiomyopathy [43–45]. Toth et al. reported that ER stress could induce cardiomyocyte apoptosis [46], and Luoa et al. reported that attenuation of ER stress prevents cardiac rupture and remodeling by modulating both cardiac apoptosis and fibrosis in a mouse model of MI [12]. However, the effect of FP on ER stress had not been reported. In the current study, FP treatment was shown to inhibit ER stress by reducing protein markers, GRP 78, and CHOP.

PPAR-*γ* is a transcription factor belonging to the nuclear hormone receptor superfamily. Previous studies have shown that PPAR-*γ* activation delays pathological changes associated with fibrosis [47, 48]; However, whether the cardioprotective effect of FP (e.g., anti-inflammatory, antisympathetic activation, anti-ER stress) was related to PPAR-*γ* activation has not been previously reported. In the current study, FP was shown to increase expression of PPAR-*γ* leading to concomitant reduction in inflammation, sympathetic activation, and ER stress following MI-cardioprotective effects that were reversed by GW9662.

## 5. Conclusions

In summary, this is the first study to show that FP reduces inflammation, excess sympathetic nerve activation, and ER stress in a rat model of MI. Because inflammatory cytokine release, sympathetic activation, and ER stress contribute to the clinical etiology of myocardial remodeling and heart failure, further studies evaluating the effects of novel interventions such as FP are warranted.

## Figures and Tables

**Table 1:** Primer sequences used in quantitative real-time PCR.

Targets	Primers	Sequences
CHOP	Forward:	5-CACTCTTGACCCTGCTTCTC-3 -3′
	Reverse:	5′-TCTTCCTCCTCTTCCTCCTG -3′
GRP78	Forward:	5′-GCACAGACGGGTCATTCCAC -3′
	Reverse:	5′–CCTATGTCGCCTTCACTCC -3′
PPAR*γ*	Forward:	5′- TGATATCGACCAGCTGAACC-3′
	Reverse:	5′- GTCCTCCAGCTGTTCGCCA-3′
TNF-*α*	Forward:	5′--GCCACCACGCTCTTCTGTC-3′ 5′-GCTACGGGCTTGTCACTCG-3′
IL-1*β*	Forward:	5′-GGGATGATGACGACCTGC-3′
	Reverse:	5′-CCACTTGTTGGCTTATGTT-3′
GAPDH	Forward:	5′- AATGCATCCTGCA CCACCA A -3′
	Reverse	5′-GATGCCATAT TCATTGT CATA–3′

**Table 2:** Effect of FP on myocardial histopathology and cardiac function.

	Sham	MI	MI + FP	FP	MI + FP + GW9662	MI + GW9662
Infarct size (%)		47.2 ± 0.7	45.6 ± 0.9		45.3 ± 0.5	47.6 ± 0.4
Infarction wall thickness (mm)		0.41 ± 0.04	0.72 ± 0.06^#^		0.46 ± 0.03^&^	0.42 ± 0.04
Body weight (g)	289 ± 4	226 ± 6^*∗*^	279 ± 3^#^	302 ± 5	241 ± 3^&^	332 ± 4
Lung wet/dry wet ratio	3.90 ± 0.21	5.01 ± 0.31^*∗*^	4.37 ± 0.25^#^	3.84 ± 0.17^#^	4.96 ± 0.27^&^	4.92 ± 0.27
Liver wet/dry wet ratio	2.35 ± 0.05	2.99 ± 0.04^*∗*^	2.49 ± 0.05	2.41 ± 0.06	2.76 ± 0.09^&^	2.94 ± 0.10
LVID (d) (mm)	6.71 ± 0.29	9.85 ± 0.30^*∗*^	7.79 ± 0.38^#^	6.59 ± 0.25	9.55 ± 0.23^&^	9.47 ± 0.23
LVID (s) (mm)	3.27 ± 0.31	8.65 ± 0.41^*∗*^	5.24 ± 0.28^#^	3.55 ± 0.29	8.50 ± 0.34^&^	3.16 ± 0.38
LVEF (%)	68 ± 2.44	37 ± 3.88^*∗*^	56 ± 2.74^#^	69 ± 2.22	39 ± 3.12^&^	38 ± 3.66
LVFS (%)	37 ± 2.20	20 ± 3.03^*∗*^	31 ± 2.59^#^	37 ± 2.45	22 ± 2.84^&^	20 ± 2.70

LVID (d), left ventricular internal dimension in diastole; LVID (s), left ventricular internal dimension in systole; LVEF, left ventricular ejection fraction; LVFS, left ventricular fraction shortening. MI, myocardial infarction. Values are means ± SEM; ^*∗*^*P* < 0.05 compared with the Sham group;^#^*P* < 0.05 compared with the MI group; ^&^*P* < 0.05 versus the MI + FP group.

## Data Availability

The datasets generated and analyzed during the present study are available from the corresponding author on reasonable request.
